# Deficiency in four and one half LIM domain protein 2 (FHL2) aggravates liver fibrosis in mice

**DOI:** 10.1186/1471-230X-13-8

**Published:** 2013-01-14

**Authors:** Sebastian Huss, Christian Stellmacher, Diane Goltz, Inna Khlistunova, Alexander C Adam, Jonel Trebicka, Jutta Kirfel, Reinhard Büttner, Ralf Weiskirchen

**Affiliations:** 1 Institute of Pathology, University of Cologne, Cologne, Germany; 2 Institute of Clinical Chemistry and Pathobiochemistry, RWTH University Hospital Aachen, Aachen, Germany; 3 Institute of Pathology, University of Bonn, Bonn, Germany; 4 Department of Internal Medicine I, University of Bonn, Bonn, Germany; 5 Gerhard-Domagk-Institute of Pathology, University of Münster, Münster, Germany

## Abstract

**Background:**

Four and one half LIM domain protein 2 (FHL2) has been reported to be a key regulator in many cellular processes being associated with fibrogenesis such as cell migration and contraction. Moreover, hepatic FHL2 is involved in regulation pathways mediating proliferation and cell death machineries. We here investigated the role of FHL2 in the setting of experimental and clinical liver fibrosis.

**Methods:**

FHL2^−/−^ and wild type (wt) mice were challenged with CCl_4_. Fibrotic response was assessed by quantitative real time PCR (qRT-PCR) of fibrotic marker genes, measurement of hydroxyproline content and histological methods. Murine FHL2^−/−^ and hepatic stellate cells (HSC) were isolated and investigated *via* immunofluorescence. Human fibrotic and normal liver samples were analysed immunohistochemically using antibodies directed against FHL2.

**Results:**

FHL2^−/−^ mice displayed aggravated liver fibrosis compared to wt mice. However, immunofluorescence revealed no significant morphological changes in cultured FHL2^−/−^ and wt myofibroblasts (MFB). In human liver samples, FHL2 was strongly expressed both in the nucleus and cytoplasm in MFB of fibrotic livers. In contrast, FHL2 expression was absent in normal liver tissue.

**Conclusions:**

Deficiency of FHL2 results in aggravation of murine liver fibrosis. In human liver samples, FHL2 is expressed in activated HSCs and portal fibroblasts in human fibrotic livers, pointing to a central role of FHL2 for human hepatic fibrogenesis as well.

## Background

Liver fibrosis is characterized by a significant increase of collagenous matrix. Complete organ fibrosis represents the final course of several chronic progressive liver diseases and end-stage liver disease is associated with a high morbidity and mortality. Myofibroblastic activated HSC represent the key fibrogenic cells in the liver. Besides the production of extracellular matrix, these cells show increased proliferation, migration and contraction. To date, hepatic fibrosis is seen as a complex wound healing process, not being fully understood [1].

FHL2 belongs to the superfamily of LIM domain proteins, which harbour a common zinc finger domain allowing flexible protein-protein interaction [2,3]. Depending on the number of LIM-domains, five subfamilies are to be distinguished [4]. FHL2 belongs to the family of four an a half LIM domain proteins. This family is known to interact with integrins at the plasma membrane and function as transcriptional coactivator after nuclear translocation [5]. FHL2 has been shown to play an active part in fibrogenesis both in wound healing and tumor stroma. In line, it was found that mice that are deficient for FHL2 are characterized by a delayed healing of skin wounds and an overall reduced transcriptional activation of smooth muscle actin and reduced contractility of MFB [6]. In addition, it was reported that FHL2-deficiency leads to disturbed intestinal wound healing [7]. Most recently, Gullotti et al. observed that FHL2 activation and nuclear transactivation occurs much more intensively in sporadic colon cancers than in microsatellite-instable, hereditary colon cancer and correlates with intensive tissue remodelling and advanced metastasis [8]. Furthermore, transgenic mice constitutively overexpressing FHL2 in the liver were shown to have a significantly higher proliferation rate that was associated with concomitant apoptosis resulting in normal liver mass suggesting that FHL2 is a crucial gatekeeper that controls fundamental hepatic processes and liver diseases [9].

As FHL2 has been shown be involved in different forms of fibrogenesis and transdifferentiation of cells into a MFB-like, contractile cell phenotype, we aimed at investigating whether FHL2 is involved in hepatic fibrogenesis as well.

## Methods

### Animals

Male C57BL/6 mice (Harlan Laboratories, Eystrup, Germany) aged 10–12 weeks were kept under controlled environmental conditions with a 12-h light–dark cycle. Mice were fed on a standard laboratory diet with food and water *ad libitum*. FHL2^−/−^ knockout mice were bread into C57Bl/6 background for more than ten generations. All animal experiments were approved by the *Landesamt für Natur, Umwelt und Verbraucherschutz NRW (LANUV)*, Recklinghausen, Germany under reference number 8.87-51.04.20.09.

### Induction of liver fibrosis

Six to eight weeks old wt mice (n = 9) or FHL2-deficient mice (n = 12) weighing approximately 18–20 g received intraperitoneal injections twice weekly of 0.7 ml/kg body weight of CCl_4_ in equal volume of mineral oil for up to 6 weeks (i.e. a total of 12 i.p. injections), whereas mineral oil alone was used as controls in wt animals (n = 4) or FHL2-deficient mice (n = 4).

### Tissue collection

After 6 weeks of treatment, the animals were sacrificed by cervical dislocation. The livers were removed and samples snap-frozen in liquid nitrogen and stored at −80°C or fixed in formaldehyde (4%). Blood was drawn from the right ventricle, centrifuged and the serum was stored at −80°C until further analysis.

### Measurement of serum parameters

Blood biochemical parameters (bilirubin, ALT, AST and total protein) were measured using the Modular Pre-Analytics (MPA) system (Roche Diagnostics, Mannheim, Germany).

### Histology and immunohistochemistry

After 24 hours fixation in 4% buffered formalin, the liver specimens were embedded into paraffin. Histological quantitative examination of liver fibrosis was performed on sections after standard Sirius Red staining. For the morphometric analysis of Sirius Red-stained specimens, at least 10 mm^2^ of liver tissue was analysed by means of computational analysis (Histoquant, 3DHistech, Budapest, Hungary). Large bile ducts and vessels were excluded. The principle of computational analysis has been described elsewhere [10,11]. For the analysis of activated HSC in wt and FHL2^−/−^ livers, paraffin liver sections were co-stained with antibodies for collagen III (anti-collagen III, Biozol/SouthernBiotech, Eching, Germany) and α-SMA (anti-α-SMA, clone E184, Biomol/Epitomocs, Hamburg, Germany).

### Hepatic hydroxyproline content

Hepatic hydroxyproline content was determined in analogue segments (50 mg) of snap-frozen livers using standard methods. The results are calculated as mg/g of wet liver tissue.

### Quantitative RT-PCR

RNA isolation, reverse transcription with 0.5 μg total RNA, and detection by RT-PCR were performed as previously described [12]. Most of the primers and probes for RT-PCR were obtained as a ready-to-use mix (α-SMA, Fibronectin, TGF-β, Procollagen Iα, from Applied Biosystems, Foster City, USA). Murine GFAP was amplified using primers mGFAPf (5^′^-CCT TCT GAC ACG GAT TTG GT-3^′^) and mGFAPr (5^′^-ACA GAC TTT CTC CAA CCT CCA G-3^′^) and murine fibulin-2 was amplified using primers mFbln2f (5^′^-CCA TCA AAC ACT CGT CTT GGT-3^′^) and mFbln2r (5^′^-TGT TGT TGG GGA CAC AGC TA-3^′^), respectively. GAPDH served as endogenous control (primers and probes ready-to-use mix by Applied Biosystems). RT-PCR (ABI 7300 sequence detector) and PCR reaction (2x TaqMan-PCR-mastermix, Applied Biosystems) were performed as previously described [12]. For each of the genes, a validation experiment was performed. Efficiencies of RT-PCR for the target gene and the endogenous control were approximately equal. -ΔCT expresses the difference between number of cycles (CT) of the target genes and the endogenous control. Results were expressed as 2-ΔΔCt, and express the x-fold increase of gene expression compared to control group.

### Cell culture

Primary HSC from wt and FHL2 deficient mice were isolated by pronase-collagenase perfusion following by FACS-based purification protocols [13]. 10 × 10^4^ cells each were seeded onto glass coverslips and cultivated in Dulbecco’s modified Eagle medium (Lonza BioWhittaker, Verviers, Belgium) supplemented with 10% FCS (PAA Laboratories, Pasching, Austria), 4 mM L-Glutamine (Lonza), 100 U/ml penicillin and streptomycin (Lonza). For generation of fully transdifferentiated MFB, the cells were passaged once and cultured for an additional 2 or 7 day period. At indicated time points, the cells were fixed in 4% paraformaldehyde for 10 min and stained with a rhodamin phalloidin.

### FHL2 immunohistochemistry in human liver fibrosis

We investigated the expression of FHL2 immunohistochemically in three samples of human liver fibrosis. Normal liver served as control. The staining procedure was carried out as described earlier [8]. In brief, immunohistochemistry was performed on 4-μm-thick paraffin-embedded sections by use of the peroxidase-conjugated avidin–biotin method. Primary antibodies included FHL2 (kind gift of R. Schüle, University Hospital Freiburg, Freiburg, Germany) and α-SMA (clone 1A4, DAKO, Hamburg, Germany, 1:4000).

### Statistical analysis

Data are presented as mean±standard error of the mean (SEM). Student’s t-test was used for comparison where appropriate, p-values < 0.05 were considered statistically significant.

## Results

### Hepatocellular injury after CCl_4_-treatment

**Figure 1:**
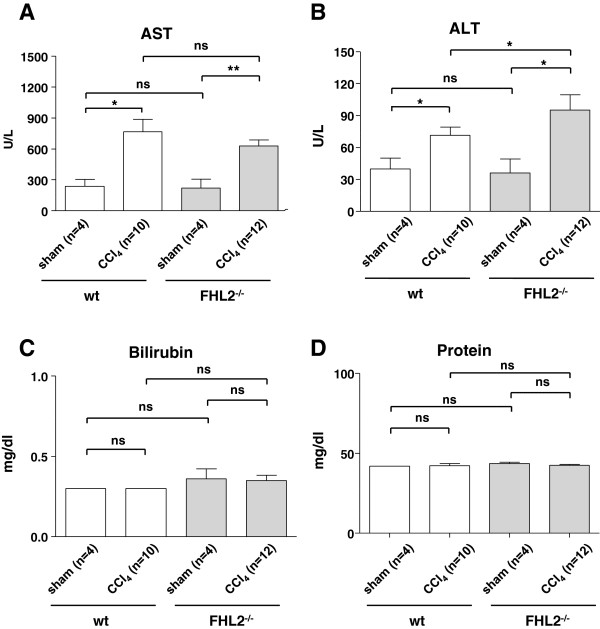
**Serum analysis in wild-type and FHL2-deficient mice after CCl**_**4 **_**challenge.** Serum analysis in wt and FHL2-deficient mice after CCl_4_ challenge. Biochemical markers were investigated in serum samples of wt or FHL2 deficient mice that received CCl_4_ or mineral oil and are shown for (**A**) aspartate transaminase (AST), (**B**) alanine aminotransferase (ALT), (**C**) bilirubin, and (**D**) total protein.

The aminotransferases ALT and AST were significantly increased after CCl_4_ treatment in both wt and FHL2^−/−^ mice compared to their sham treated littermates (Figure 1, upper panels). Significant changes were noted between CCl_4_-challenged wt and knockout mice concerning ALT, but not AST. Bilirubin and protein levels remained almost unchanged in all groups (Figure 1, lower panels).

### Hepatic fibrosis in mice

**Figure 2:**
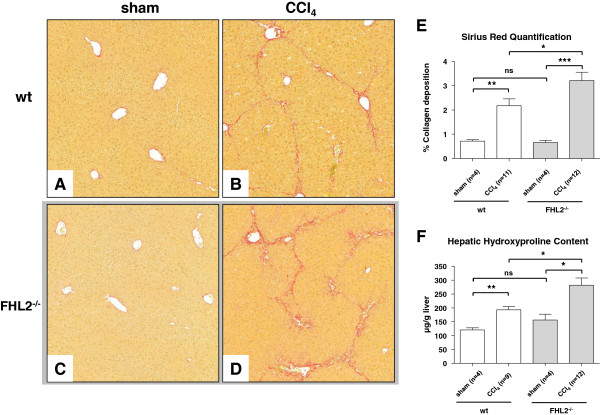
**Hepatic fibrosis in wt and FHL2-deficient mice after CCl**_**4 **_**challenge.** Liver specimens of CCl_4_-treated animals were stained with (**A-D**) Sirius Red and representative images were made using a standard light microscope (100x). Relative collagen deposition was further quantified by (**E**) densitometry or by measuring (**F**) direct hepatic hydroxyproline content. Non significant differences (ns), significant differences (*), and highly significant differences (**-***) are marked.

**Figure 3:**
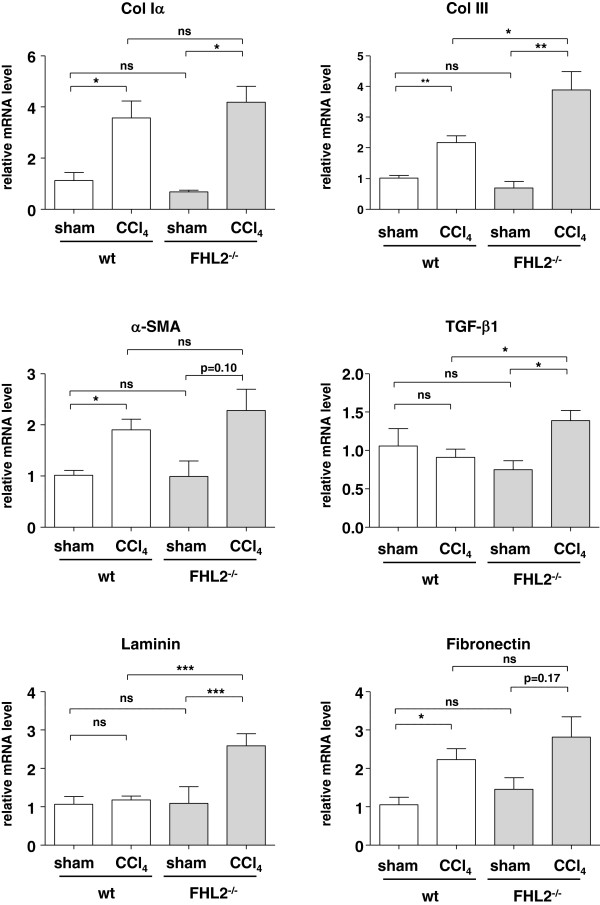
**Expression analysis of genes associated with fibrogenesis in mice subjected to CCl**_**4 **_**challenge.** RNA was isolated from livers of wt or FHL2 deficient mice that received CCl_4_ or mineral oil and analyzed for expression of collagen type αI, collagen type III, α-SMA, TGF-α, laminin and fibronectin by qRT-PCR analysis. Non significant differences (ns), significant differences (*), and highly significant differences (**-***) are indicated.

After CCl_4_-treatment, hepatic fibrosis was observed in wt and FHL2^−/−^ mice. Interestingly FHL2^−/−^ mice developed significantly aggravated liver fibrosis compared to respective wt control mice as demonstrated in Sirius Red stain (Figure 2A-2D). This finding was also confirmed by both morphometric analysis of Sirius Red stained histological slides (Figure 2E) and measurement of hepatic hydroxyproline content as the main amino acid of collagen (Figure 2F). After CCl_4_ challenge, the lack of FHL2 further resulted in an increased expression of collagen type III, TGF-β1, and laminin (Figure 3), while the mRNA quantities of Collagen type I, α-SMA and fibronectin were not statistically different between control and FHL2 deficient mice.

### Hepatic stellate cell activation in mice

**Figure 4:**
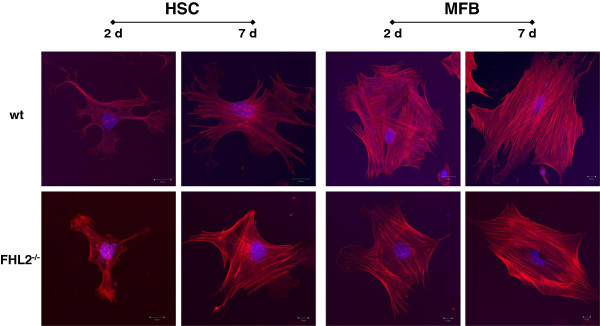
**Transdifferentiation of isolated HSC to MFB.** HSC from wt and FHL2 deficient mice were isolated by FACS-based protocols and plated on plastic surfaces for 2 or 7 days. For generation of fully transdifferentiated MFB, the cells were passaged once and cultured for an additional 2 or 7 day period. At indicated time points, the cells were fixed in 4% paraformaldehyde and stained with a rhodamine-phalloidin-conjugate. Note the typical appearance of F-actin fibres in both cell phenotypes that are highly characteristic for activated HSC and transdifferentiated MFB.

To test if HSC isolated from wt and FHL2 deficient show a different tendency to become activated in culture, we isolated primary HSC from respective animals using established procedures. The typical appearance of F-actin fibres in both cell phenotypes was noted that is highly characteristic for activated HSC and transdifferentiated MFB. However, no significant differences between the two genotypes were noted suggesting that the gene deficiency does not interfere with cellular activation or transdifferentiation *per se* (Figure 4).

**Figure 5:**
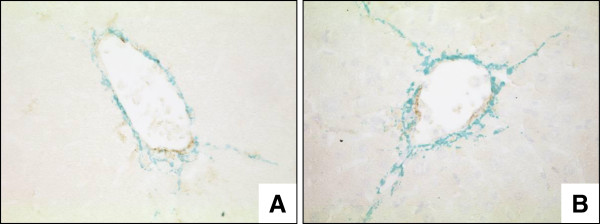
**Collagen III/α-SMA expression in wt and FHL2-deficient mice after CCl**_**4 **_**challenge.** Compared to (**A**) wt littermates, (**B**) FHL2-deficient mice showed a stronger collagen III (*green*) expression after CCl_4_ challenge (400x). However, there was no obvious difference in α-SMA (*brown*) expression.

**Figure 6:**
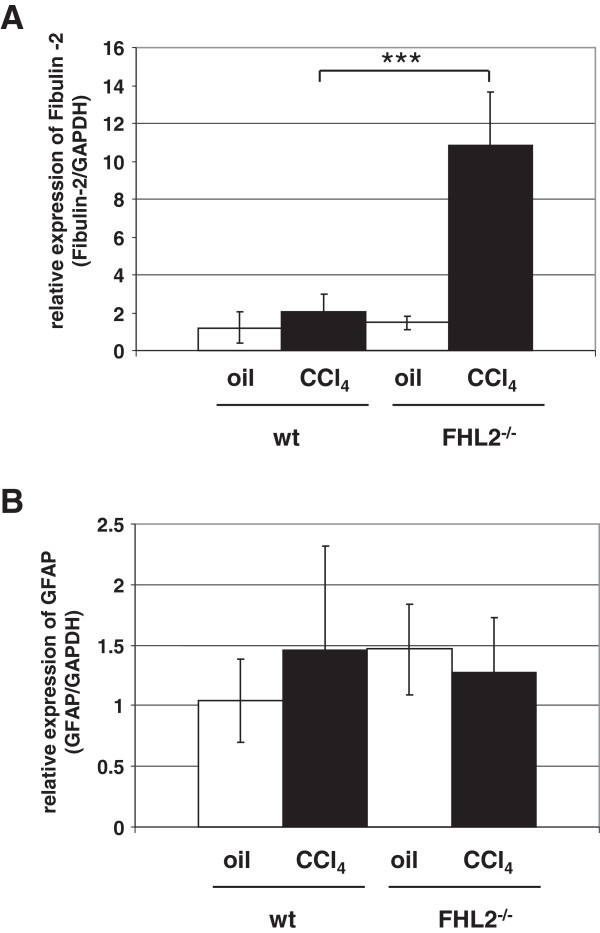
**Expression of Fibulin-2 and GFAP in wt and FHL2-deficient mice subjected to CCl**_**4 **_**challenge.** Expression of (**A**) Fibulin-2 (marker of portal fibroblasts) and (**B**) GFAP (marker of HSC) was analysed in wt and FHL2 deficient mice after CCl_4_ challenge. Animal numbers analysed in this experiment were: n = 4 (wt, oil), n = 11 (wt, CCl_4_), n = 4 (FHL2^−/−^, oil), and n = 12 (FHL2^−/−^, CCl_4_). Please note that the differences in fibulin-2 expression between wt and FHL2^−/−^ animals after CCl_4_ challenge are highly significant (***).

To further analyze the expression pattern of activated HSC in wt and FHL2 deficient livers, paraffin liver sections were co-stained with antibodies specific for collagen III and α-SMA. In this analysis, FHL2-deficient mice revealed a stronger collagen III expression after CCl_4_ challenge compared to wt animals (Figure 5). However, there were again no significant differences in α-SMA expression (not shown). However, the expression of Fibulin-2 representing a marker of portal fibroblasts [14,15] was extensively increased at the mRNA level in FHL2 deficient mice subjected to CCl_4_ treatment (Figure 6A) and expression of GFAP representing a marker of HSC was more or less the same (Figure 6B).

### FHL2 expression in human liver fibrosis

**Figure 7:**
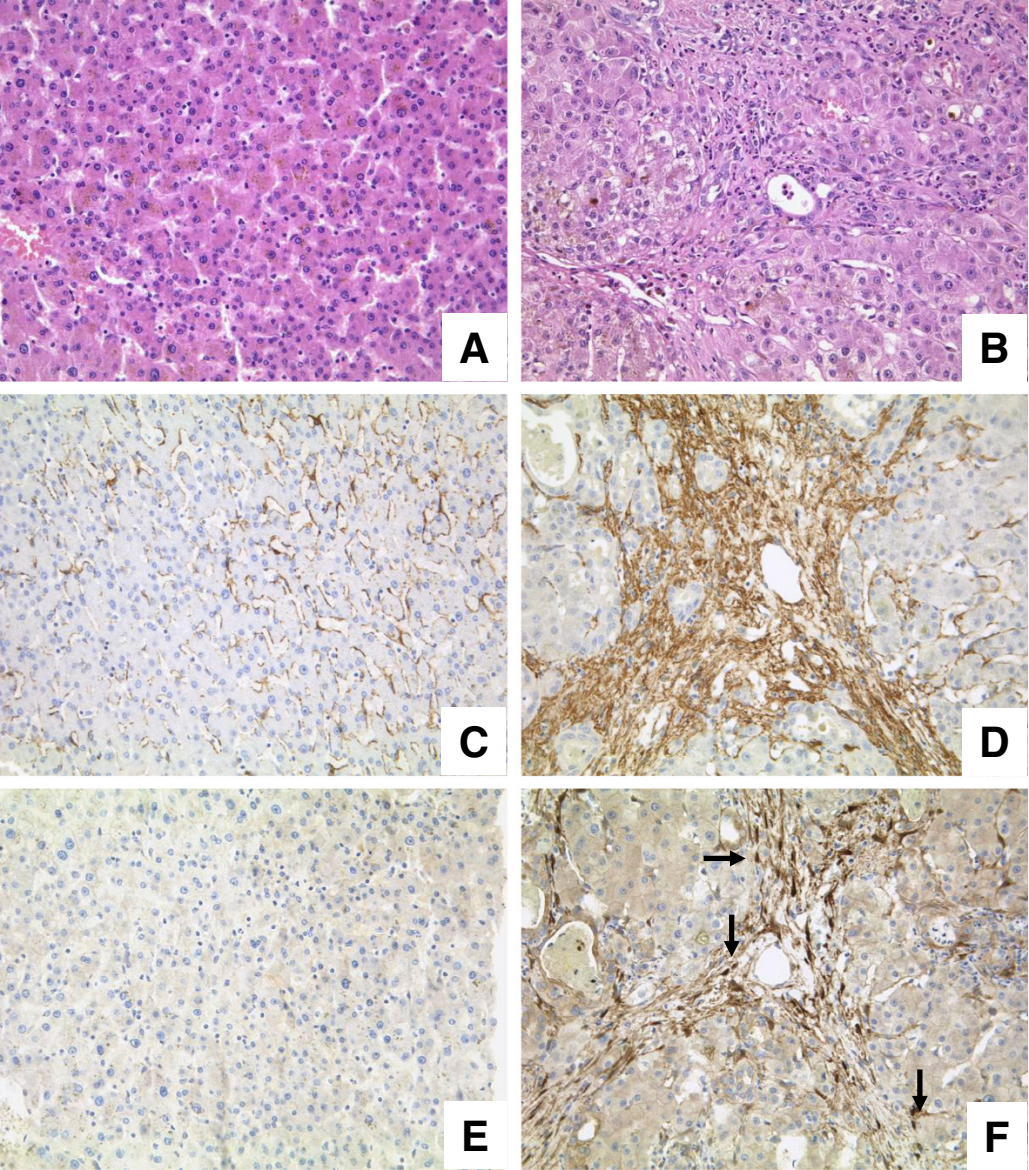
**FHL2 expression in normal and fibrotic human liver samples.** Normal (**A**, 200x, HE) and fibrotic liver samples (**B**, 200X, HE) were investigated using antibodies against α-SMA and FHL2. In normal liver, α-SMA expression was low (**C**, 200x), whereas in fibrotic liver samples a strong reactivity was found especially in activated MFB (**D**, 200x). FHL2 expression was negative in normal liver (**E**, 200x). In contrast, MFB revealed strong nuclear (arrows) as well as cytoplasmatic staining in fibrotic liver samples (**F**, 200x).

We investigated FHL2 expression in normal and fibrotic liver samples (Figure 7). In normal healthy liver, FHL2 staining was nearly absent. Stromal cells occasionally revealed weak nuclear FHL2 expression. In fibrotic liver, however, MFB and portal fibroblasts showed a strong nuclear and cytoplasmatic expression of FHL2 in fibrotic septa. A similar staining pattern was observed in cultured murine HSC undergoing transdifferentiation (not shown).

## Discussion

This study shows for the first time that FHL2 is crucial in experimental liver fibrosis. When we challenged FHL2-deficient mice with CCl_4_, we observed that FHL2-deficient mice displayed aggravated liver fibrosis compared to wt mice as indicated by morphometric analysis of Sirius Red-stained histological slides and direct measurement of hepatic hydroxyproline content in liver protein extracts that is indicative for deposited collagen. Therefore, our findings suggest a protective mechanistic role of FHL2 during hepatic fibrogenesis.

Kirfel et al. reported impaired intestinal wound healing in FHL2-deficient mice. They identified disturbed collagen type III production being responsible for reduced mechanical stability of experimental intestinal anastomoses. They concluded that FHL2 is a critical factor regulating collagen expression in the early phase of wound healing.

In normal liver, collagen type I and III are found in portal tracts and around hepatic veins. During liver fibrosis, excess collagen type I and III are deposited not only in portal tracts but also in the lobule leading to major architectural changes. In the present study aggravated liver fibrosis was especially associated with upregulated collagen III expression in mice lacking FHL2 and subjected to CCl_4_-treatment. Interestingly, these findings are different than those observed in intestinal wound healing possibly reflecting the different biological behaviour of different cell entities involved in fibrogenesis in different organs. Concerning different cell types, FHL2 has been shown to interact as a cofactor regulating a variety of target genes. In ovarian granulosa cells, FHL2 was found to be a coactivator of NR5A nuclear receptors [16]. In prostate cancer, FHL2 acts as a coactivator, shifts to the nucleus and subsequently activates FHL2- and androgen receptor-dependent genes [17]. In our human liver fibrosis samples studied, MFB showed a strong nuclear and cytoplasmatic expression of FHL2. We therefore hypothesize, that FHL2 acts probably as a coactivator regulating important target genes in the setting of liver fibrosis as well.

Park et al. showed that FHL2 is involved in bundling of focal adhesion structures and in proper allocation of matrix proteins [18]. In our experiments, fibronectin was upregulated in both wt and FHL2^−/−^ mice (Figure 3), whereas laminin was only strongly upregulated in FHL2 deficient mice and not in wt mice. However, we have not analyzed if the difference in laminin expression is directly involved in FHL2 functionality during hepatic fibrogenesis, other LIM domain proteins such as the LIM only protein Hic-5 are also known to influence laminin expression [19]. Although we have actually no further data that highlight the activity of FHL2 in laminin expression, it will be mandatory to clarify if this regulation is a general attribute of individual classes of LIM domain proteins.

The recent finding demonstrating that FHL2 controls liver homeostasis by co-ordinately regulating cell proliferation and cell death in hepatocytes [8] and our findings demonstrating that FHL2 might be an essential factor in controlling hepatic fibrogenesis *via* regulating HSC activation and transdifferentiation. It further suggests that this LIM domain in liver is multi-functional impacting crucial processes involved in control of liver homeostasis. However, we could not detect a difference in HSC transdifferentiation *in vitro* (Figure 4), either suggesting that (i) the gene deficiency induces only differences *in vivo* or that (ii) the antifibrotic functionality of FHL2 is more pronounced in portal fibroblasts. In our view, the second possibility is more likely since the expression of fibulin-2 representing a marker of fibrogenic portal fibroblasts is highly significant upregulated in FHL2 deficient mice when subjected to CCl_4_ challenge (Figure 6A) , while the expression of GFAP correlating with HSC numbers is the same in FHL2 deficient and control animals under these conditions (Figure 6B).

## Conclusions

We show that deficiency of FHL2 results in aggravation of murine liver fibrosis. In human liver samples, FHL2 is expressed in activated HSCs and portal fibroblasts in human fibrotic livers, pointing to a central role of FHL2 for human hepatic fibrogenesis as well. Altogether, these findings indicate that this LIM domain protein is most relevant when liver homeostasis is affected.

## Competing interests

The authors declare no competing interests.

## Authors’ contributions

SH performed the animal experiments drafted the manuscript and analyzed the histological samples. DG helped with the animal experiments. CS and IK performed the *in vitro* experiments and PCR. AA analyzed the human liver samples. JT performed the hydroxyproline assay. JK and RB supervised the animal experiments and participated in study design. RW is responsible for the serum analysis and was involved in the study design as well as in drafting the manuscript. All authors carefully read and approved the final manuscript.

## Pre-publication history

The pre-publication history for this paper can be accessed here:

http://www.biomedcentral.com/1471-230X/13/8/prepub
